# ﻿Genetic and morphological evidence support the specific status of the endemic *Ericaandevalensis* (Ericales, Ericaceae)

**DOI:** 10.3897/phytokeys.244.120914

**Published:** 2024-07-05

**Authors:** Iván Rodríguez-Buján, Pilar Díaz-Tapia, Jaime Fagúndez

**Affiliations:** 1 Universidade da Coruña, BIOCOST research group, Centro Interdisciplinar de Química e Bioloxía (CICA), Rúa As Carballeiras, 15071, A Coruña, Spain; 2 Universidade da Coruña, Facultade de Ciencias, Departamento de Bioloxía, 15071, A Coruña, Spain; 3 Universidade de Santiago de Compostela, Facultade de Bioloxía, Departamento de Botánica, 15072 Santiago de Compostela, Spain

**Keywords:** Endemic species, *Erica*, metallophyte, population structure, species delimitation, systematics

## Abstract

Assessing the taxonomic status of closely related taxa is crucial in plant systematics and can have important implications for conservation and human plant use. *Ericaandevalensis* Cabezudo & Rivera is a metallophyte endemic species from highly metal-polluted soils of SW Iberian Peninsula, an area with a mining history going back more than 5,000 years. *Ericaandevalensis* is closely related to *Ericamackayana* Bab., a northern Iberian species also present in western Ireland. The status of *E.andevalensis* as a species or subspecies subordinated to *E.mackayana* is subject to debate. Here, we assessed the genetic and phenotypic relationship between both species, including the population structure of *E.andevalensis*. We used high throughput sequencing to determine genome-wide Single Nucleotide Polymorphisms (SNPs), and morphometric analyses from 35 reproductive and vegetative traits. The morphological analysis showed at least eight characters that can discriminate the two species, from which ovary hairiness and the size of leaf glandular hairs were the most informative. Genetic analyses showed that each species formed a monophyletic cluster with full support, separated by an interspecific genetic distance >4-fold higher than intra-specific distance. Population genetic analyses of *E.andevalensis* shows that populations are highly structured, with the Portuguese one as the most isolated and less variable. These results support the recognition of *E.andevalensis* as a distinct species with a highly constrained ecological requirements and a narrow geographic distribution, but with a limited gene flow between populations. We discuss the implications of these outcomes in conservation policies and potential uses of *E.andevalensis* such as decontamination of polluted soils.

## ﻿Introduction

The species is the fundamental unit in taxonomy and systematics of living organisms (De Queiroz 2005). This perspective extends to all fields of biology, leading to considerable scientific effort being dedicated to this taxonomic category. Beyond a scientific perspective, species delimitation is relevant in conservation planning and biodiversity assessment of protected areas (Coates et al. 2018; Galtier 2019).

In plant taxonomy, morphological characters have historically been the primary source of evidence for delimitating species (Whittemore 1993; Soltis and Soltis 2012). However, the more recent application of DNA data to assess species diversity has significantly impacted the field of taxonomy. Ideally, species delimitation would be informed by a comprehensive understanding of variability and population structure, as well as knowledge of the connections among closely related lineages (Galtier 2019). Since most species exhibit geographic variation, it is important to obtain a good representation of the distribution area to distinguish true discontinuities, which could imply lineage separation, from within-lineage variation (De Queiroz 2007). In lineages with incipient separation, considering other lines of evidence (e. g. ecological, morphological, and/or phenological) in addition to molecular data provides the basis for a more robust integrative taxonomy (Pante et al. 2015).

High throughput sequencing (HTS) is the most significant recent advance in molecular techniques, as it greatly facilitates the generation of large amounts of DNA data. Restriction site-associated DNA (RAD-seq) and similar approaches involve enzymatic fragmentation of genomic DNA coupled with HTS to determine large numbers of molecular markers with genome-wide coverage (Andrews et al. 2016). The enhanced resolution provided by these techniques has been applied to disentangle complex relationships between closely related species as well as to study the genetic structure of populations (e.g. Pante et al. 2015; Feng et al. 2018).

*Erica* is among the largest genera of seed plants, with 851 accepted species (Elliott et al. 2024; Oliver et al. 2024; WFO 2024), most of them concentrated in the Cape Floristic Region (Manning and Goldblatt 2012). However, the genus most probably originated in the Palearctic region, where only 23 species live today (Mugrabi de Kuppler et al. 2015). Despite the relatively low number of *Erica* species in Europe, they hold a large phylogenetic diversity compared to the species-rich Cape floristic region (Pirie et al. 2024). *Erica* also plays an important ecological role as dominant species in different European habitats (Fagúndez 2013). European heathlands are facing many threats, and their habitats are decreasing, resulting in the isolation of populations and local extinctions (Fagúndez 2013). Understanding the genetic diversity and population structure of these species becomes essential to assess the conservation status of their populations and infer their future survival in a context of global change (Frankham 2010).

**Figure 1.**
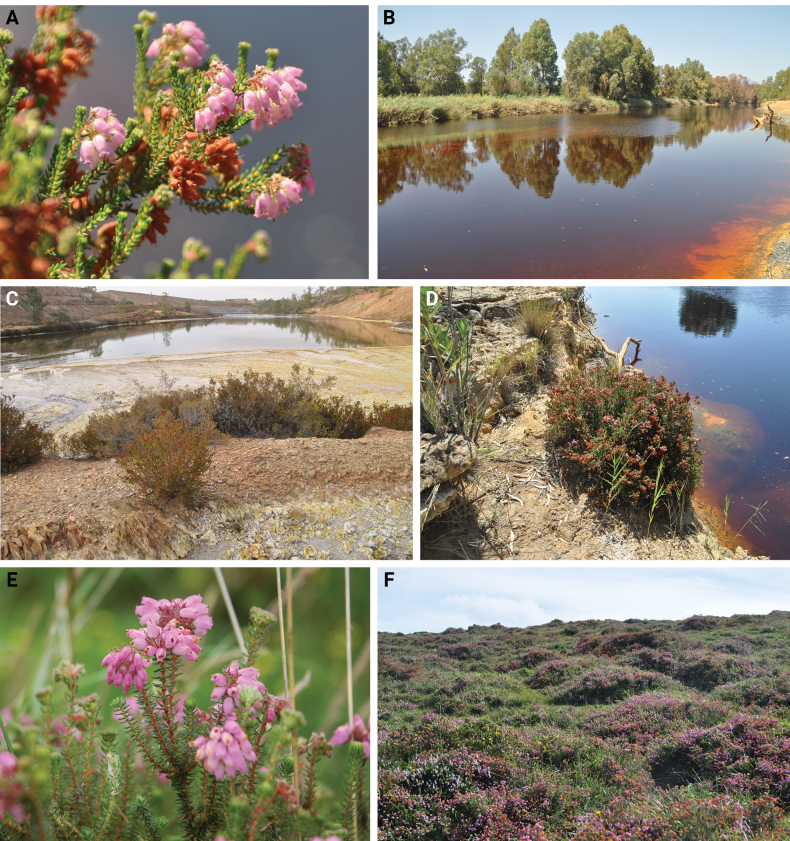
**A** Flowering stems of *Ericaandevalensis***B** Tinto river shores in Huelva, SW Spain, the habitat of *E.andevalensis***C** São Domingos, Portugal, abandoned mining area with *E.andevalensis* in front **D***Ericaandevalensis* in the shores of Odiel river **E** flowering stems of *Ericamackayana***F** wet heathland dominated by *E.mackayana* in Galicia, NW Spain.

*Ericaandevalensis* Cabezudo and Rivera, occurs in a restricted area in the south-west of the Iberian Peninsula, mainly in western Andalusia, Spain, and bordering Portugal (Cabezudo and Rivera 1980; Buira et al. 2017). It colonizes wet soils within the Iberian Pyrite Belt, characterized by high levels of sulfur and heavy metals. It frequently forms nearly monospecific communities along polluted riversides and in abandoned mines (Aparicio 1999; Fig. 1). As a restricted metallophyte endemic species, *E.andevalensis* is considered a threatened species (BOJA 2012; Carapeto et al. 2020).

*Ericaandevalensis* is closely related to *E.mackayana* and both species are morphologically similar. However, they have disjunct distributions, as *E.mackayana* is restricted to the northern Iberian Peninsula (Mugrabi de Kuppler et al. 2015; Fagúndez and Díaz-Tapia 2023). Despite morphological similarities, both species can be distinguished by the pattern and size of glandular hairs and the presence/absence of other small non glandular hairs (Cabezudo and Rivera 1980). However, some variability was found in these characters and both species can lack glandular hairs. As a result, *E.andevalensis* has been considered a subspecies under *E.mackayana* (McClintock and Nelson 1989), although Nelson (2012) subsequently treated *E.andevalensis* as a species in its own right. Additional research has revealed further characters that distinguish both taxa, as they differ in seed morphology (Fagúndez and Izco 2004). Seed characters have been proven useful in delimitating *Erica* species (e.g. Szkudlarz 2009; Fagúndez and Izco 2010). Also, Bayer (1993) described small hairs in the ovary of some individuals of *E.andevalensis* that were never observed in *E.mackayana*. The discussion on the taxonomic rank of *E.andevalensis* continues (see Nelson and Small 2000; BOJA 2012; Carapeto et al. 2020; GBIF 2023; WFO 2024).

In this study, we aim to reassess the taxonomic identity of *E.andevalensis* as a distinct species from *E.mackayana* combining molecular and morphological data. By studying the whole geographic range of both species, we aim to identify morphological traits that might reliably distinguish *E.andevalensis* from *E.mackayana*. In addition, we aim to study the population structure of *E.andevalensis*, to understand the level of isolation and gene flow among populations and provide better guidelines for conservation.

## ﻿Material and methods

### ﻿Plant material

**Figure 2.**
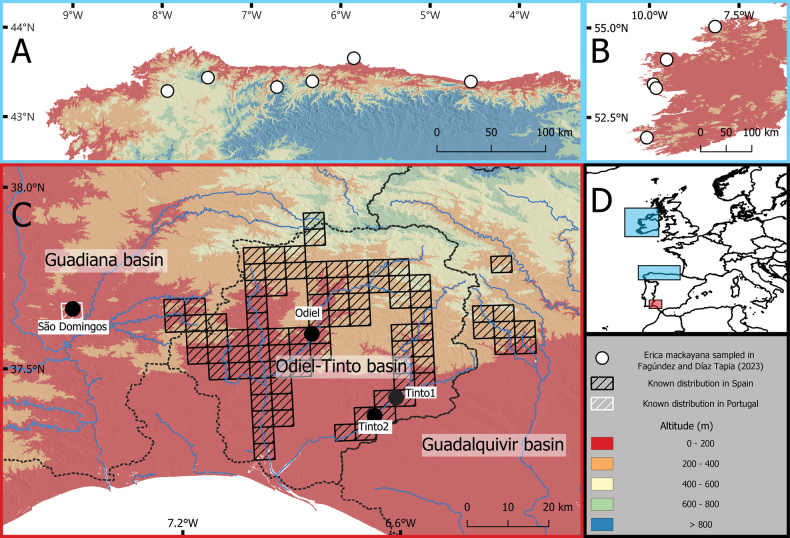
Distribution of the sampled populations of *E.andevalensis* and *E.mackayana***A***Ericamackayana* populations (as in Fagúndez and Díaz-Tapia 2023) sampled in NW Iberian Peninsula **B***Ericamackayana* populations (as in Fagúndez and Díaz-Tapia 2023) sampled in Ireland **C***Ericaandevalensis* populations sampled. The distribution of *E.andevalensis* is represented in a 5×5 UTM grid from https://www.ideandalucia.es/catalogo/inspire/srv/api/records/625a6b54-bfc1-4589-8571-b4503bf262c2 (Spain) and Carapeto et al. (2020) (Portugal) **D** location of the three studied regions in Europe.

We sampled 38 plants from four populations of *E.andevalensis* in August 2021. Seventy-one plants from ten populations of *E.mackayana* analysed in Fagúndez and Díaz-Tapia (2023) were also included in the analyses (Fig. 2, Suppl. material 1: table S1). Additionally, one *E.tetralix*, the closest species to the *E.mackayana-andevalensis* clade (Mugrabi de Kuppler et al. 2015), was included as outgroup in the phylogenetic analyses.

A subsample of each specimen was dried in silica gel, and the remaining material was prepared for morphological studies. All herbarium vouchers were deposited in SANT herbarium.

### ﻿Morphometric data and analyses

Each sample was mounted in a herbarium sheet and scanned at a minimum resolution of 1000 dpi. In addition, a minimum of two fresh flowers per specimen were dissected to measure ovaries and anthers. Pictures of ovaries and anthers were taken using an OLYMPUS C3040-ADU for subsequent digital analysis. For leaf micro-characters, we used an optical microscope at 40–100 magnifications. All images were analyzed using ImageJ software (Schneider et al. 2012).

**Figure 3.**
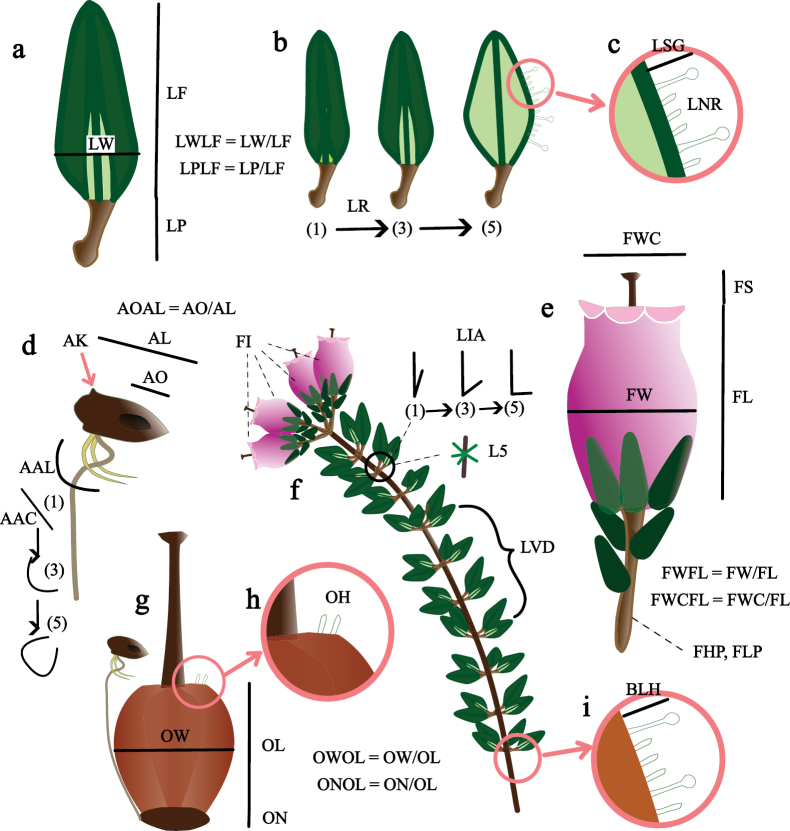
Schematic representation of the morphological traits measured in the samples of *Ericaandevalensis* and *E.mackayana*. Leaf traits (**a**) (LF = Leaf length; LW = Leaf width; LP = Petiole length), variation on leaf morphology (**b**) (LR = Leaf rolling degree), and indumentum (**c**) (LSG = length of glandular hairs; LNR = presence of non-glandular hairs). Stamen traits (**d**) (AL = Anther length; AO = Anther pore; AAL = Anther appendix length; AAC = Anther appendix curvature; AK = Anther knob), flower morphology (**e**) (FL = Corolla length; FW = Corolla width; FWC = Corolla opening; FS = Style exertion; FHP = Pedicel hairiness, FLP = Pedicel length), and arrangement of a flowering branch (**f**) (FI = Flowers per inflorescence; LIA = Leaf insertion angle, L5 = 5nate leaves, LVD = Density of whorls). The ovary (**g**) (OL = Ovary length; OW = Ovary width; ON = Nectary size) and detail of ovary surface (**h**) (OH = Ovary hairiness) and stem (**i**) (BLH = Branch longest hair). Coding, measures, and additional information in Suppl. material 1: table S2.

The final dataset for morphological analyses comprises 71 individuals of *E.mackayana* and 38 of *E.andevalensis*. In the dataset, each trait was represented by one value per specimen, thus the arithmetic mean was calculated for multiple measures (Suppl. material 1: table S2). We measured a total of 35 traits including 24 quantitative, 8 ordinal and 3 binary (Fig. 3, Suppl. material 1: table S2). Each trait was measured once or several times per sample. We tested for differences between the means in the two species for each variable using a non-transformed dataset. For quantitative variables, we applied t-tests or Wilcoxon rank-sum tests, depending on whether the variables met normality and homocedasticity assumptions. Ordinal variables were compared using Chi-squared tests. In the case of binary variables (LNR, L5 and LS2G), the Fisher’s exact test was conducted (Suppl. material 1: table S3).

We performed a principal component analysis (PCA) with all samples and traits. Since each trait was on a different scale, the prep function from the PCAMETHODS package (Stacklies et al. 2007) was used to normalize and center all variables. Missing data in the final matrix represented 12.08%. To overcome this issue, we used a K-Nearest-Neighbor approach from DMWR2 package (Torgo 2016). Subsequently, the FACTOEXTRA package was used to perform the PCA (Kassambara and Mundt 2020). Next steps were carried out using the MORPHOTOOLS2 package (Šlenker et al. 2022). All statistical analysis were conducted in R (R Core Team 2020).

### ﻿Molecular data

We used genomic data obtained from Fagúndez and Díaz-Tapia (2023) for *E.mackayana*, while genomic data of *E.andevalensis* was newly determined. One sample of *E.tetralix* from Fagúndez and Díaz-Tapia (2023) was included in the dataset to be used as the outgroup in phylogenetic analyses. DNA extraction, library preparation and sequencing followed Fagúndez and Díaz-Tapia (2023).

Nextera adaptors from sample reads were trimmed using BBDUK (BBMAP TOOLS v.38.79, http://sourceforge.net/projects/bbmap/) with the following parameters: ktrim = r, k = 17, hdist = 1, mink = 8, minlen = 100, ow = t, qtrim = r, trimq = 10. Quality of filter reads was checked using FastQC (v.0.11.9) (Andrews 2010). Denovo_map.pl pipeline from STACKS software v2.64 (https://catchenlab.life.illinois.edu/stacks/) was employed to denovo assembly and SNP calling.

To select the optimal parameters, a preliminary optimization step following the 80% rule (Paris et al. 2017) was conducted. Thirty percent of the samples with a minimum of two per population were selected. Reads with different lengths were allowed using –force-diff-len on ustacks module. We constructed 12 different catalogs, varying the -m (Minimum number of raw reads required to form a stack (a putative allele)), -M (Number of mismatches allowed between stacks (putative alleles) to merge them into a putative locus) and -n (Number of mismatches allowed between stacks (putative loci) during construction of the catalog) parameters. For all probes, -M = -n, but no -m, which ranged between 2 and 6. After optimization process, we created a new catalog choosing m = 4, M = 3, n = 3. Using a populations’ module, the dataset was exported to variant call format (VCF).

### ﻿Phylogenetic analysis

VCF file from STACKS was filtered with VCFTOOLS retaining the SNPs with less than 60% of missing data and those that were separated by at least 200 bp to avoid linkage disequilibrium issues. Furthermore, only variable SNPs present in a minimum of 4 samples were accepted. The VCF file was exported as interleaved phyllip using TASSEL (Bradbury et al. 2007). IQTREE v2.1-2 was used to filter invariant sites (Minh et al. 2020). Final dataset consisted of 6004 bp. MODELFINDER from WEB-IQTREE (Kalyaanamoorthy et al. 2017) was used to select the optimal model of the dataset based on Bayesian Inference Criterion (BIC). Using this approach, the best model was TVM+F+ASC+G4. Then, the final tree was calculated with this model in IQ-TREE v2.1-2 with 1000 non-parametric bootstrap replicates.

### ﻿Genetic distance analysis

To assess genetic distance between *E.andevalensis* and *E.mackayana*, we used Tamura-Nei distance (Tamura and Nei 1993) in MEGA11 (Tamura et al. 2021). We compared only populations with >2 individuals. Then, we calculated mean pairwise distances for population of each species and compared them with mean pairwise distance between both species.

### ﻿Population structure analysis

A new dataset per putative species was created. VCFTOOLS was used to remove indels, as well as filter SNPs retaining those that were present in at least 80% of samples and minor alleles were present in at least two samples. Also, to avoid linked loci, SNPs at a distance less than 500 base pairs (bp) were excluded. Both datasets were exported to plink format. The function filter_data() from SAMBAR package was used with default thresholds to detect and erase possible paralogous loci (De Jong et al. 2021). Final datasets consisted of 36 samples with 4,234 SNPs of *Ericaandevalensis* and 62 samples with 5,178 SNPs of *E.mackayana*.

Observed heterozygosity (H_o_) and expected heterozygosity (H_e_) were calculated with function filter_data() in SAMBAR, and nucleotide diversity was calculated with calc_diversity() of the same package. F_IS_ was calculated as 1-H_o_/H_e_ as in Weir and Cockerham (1984).

We further analysed the dataset of *E.andevalensis*, including a F_ST_ pairwise comparison among populations, using calc_diversity() function in SAMBAR and Bayesian population assignment (BPA). BPA probabilities were calculated using the function find_structure() in SAMBAR package. The optimal number of clusters (K) was determined using the elbow method on cross-entropy scores.

## ﻿Results

### ﻿Morphometric analyses

Twenty-eight out of 35 morphological characters measured showed statistically significant differences between *Ericamackayana* and *E.andevalensis* (Suppl. material 1: table S3).

**Figure 4.**
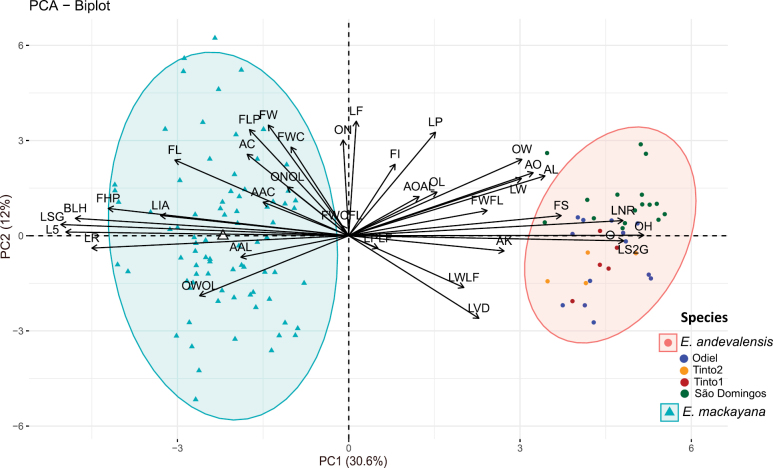
Biplot showing the first two principal components. Each ellipse represents the area that would encompass 95% of individuals assuming populations follow a normal distribution. White dots and triangles represent *E.andevalensis* and *E.mackayana* centroids respectively. *Ericaandevalensis* populations are represented in different colors: Green (São Domingos), blue (Odiel), brown (Tinto1), yellow (Tinto2). Trait acronyms as in Fig. 3 and Suppl. material 1: table S2.

**Figure 5.**
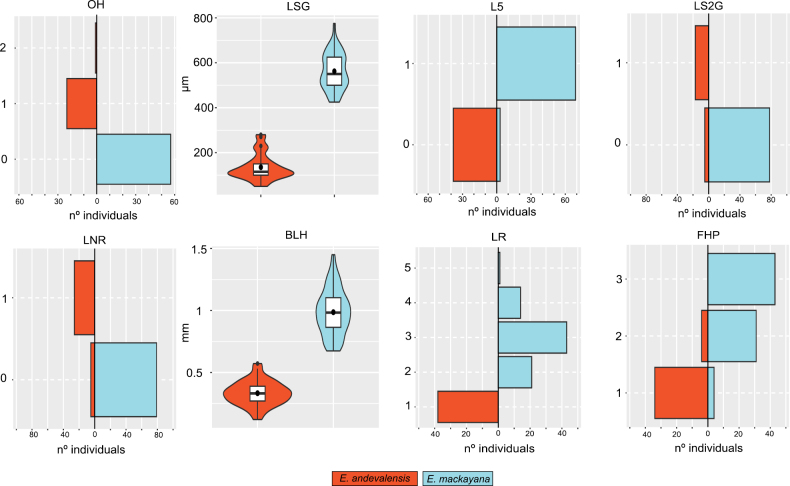
Boxplots and histograms of the traits with the highest contribution to PC1 of the PCA as in Table 1. Red corresponds to *E.andevalensis*, blue to *E.mackayana*. Black dots represent the mean. OH = Ovary hairiness; LSG = Length of glandular hairs; L5 = Presence of 5nate whorls; LS2G = Presence of more than 2 rows of glandular hairs; LNR = Presence of non-glandular hairs; BLH = Longest hair length in stem; LR = Leaf rolling degree; FHP = Pedicel hairiness.

The first and second axes of the PCA explained 30.6% and 12% of the variance in the data, respectively. PC1 clearly delimitate the two species into two separated clusters (Fig. 4). PC2 depicts intra specific variation, with the species centroids close to 0 (-0.01 in *E.mackayana* and 0.02 in *E.andevalensis* respectively). PC2 can delimitate among *E.andevalensis* populations, particularly Spanish populations (Tinto1, Tinto2 and Odiel) populations vs São Domingos from Portugal. We identified the best diagnostic characters as those with a higher value on the first PC of the PCA, thus contributing to delimitate the two species, but lower values on the second PC, which represents variability within both taxa. Eight were selected as the best diagnostic characters including ovary hairiness, length and arrangement pattern of glandular hairs in leaves, length of glandular hairs on the stem, presence of non-glandular hairs in leaves, inflorescence hairiness, leaf rolling degree and number of leaves per whorl (Table 1, Fig. 5). The contribution of the remaining variables is provided in Suppl. material 1: table S4.

**Table 1.** Selection of traits that most contribute to the two first PCA components. Percentage of variability within traits explained by the first two components. For each trait average values for each species are provided. Significance of correspondent mean differences tests is highlighted with *** if p-value <0.001. OH = Ovary hairiness; LSG = Length of glandular hairs; L5 = Presence of 5nate whorls; LS2G = Presence of more than two rows of glandular hairs; LNR = Presence of non-glandular hairs; BLH = Longest hair length in stem; LR = Leaf rolling degree; FHP = Pedicel hairiness.

Trait	Units/coding	Contribution to PC1	Contribution to PC2	*E.mackayana* mean	*E.andevalensis* mean	Significance
OH	0–5	8.584	0.000	0	1.083	***
LSG	µm	8.173	0.103	562.025	136.448	***
L5	0–1	7.890	0.011	0.958	0	***
LS2G	0–1	7.452	0.021	0	0.773	***
LNR	0–1	7.412	0.187	0	0.839	***
BLH	µm	7.369	0.246	0.986	0.333	***
LR	1–5	6.506	0.125	2.937	1	***
FHP	1–5	5.703	0.607	2.500	1.105	***

### ﻿Phylogenetic analysis

**Figure 6.**
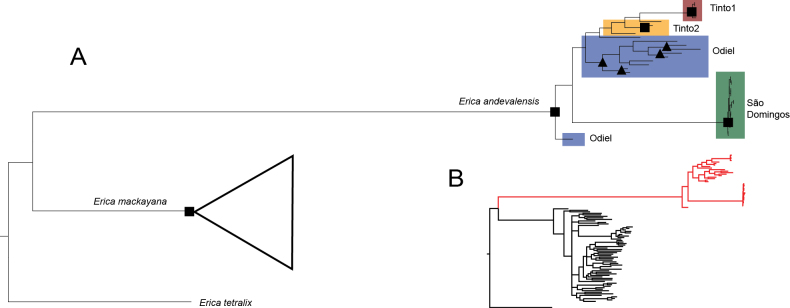
Phylogenetic tree of *E.andevalensis* and *E.mackayana***A** tree showing the *E.mackayana* clade collapsed. Colors represent sites as in Fig. 4. Bootstrap support is indicated on nodes when >80: squares represent full support and triangles 80–99% **B** expanded tree.

The phylogenetic tree placed the samples of the two species in two distinct clades that received full support (Fig. 6). Within the *E.andevalensis* clade, the samples from Tinto 1 and São Domingos formed two fully supported clades. The samples from Tinto 2 were placed as sister to Tinto 1, but this relationship was unsupported. Most samples from Odiel were placed in a moderately supported clade, except two individuals of which one was sister to the clade formed plants from Tinto 1 and 2 and the other was sister to the clade formed by all the other samples.

### ﻿Genetic distance

Nei’s genetic distance among *E.mackayana* populations was 0.280 in average, while distance among *E.andevalensis* populations was 0.268 (Suppl. material 1: table S5). Mean Nei’s genetic distance between both species was 1.146. Therefore, the inter-specific average distance between both species was >4 times greater than intra-specific.

### ﻿Populations structure of *E.andevalensis*

**Figure 7.**
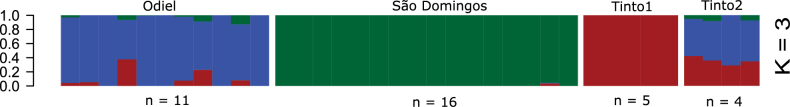
Bayesian individual assignment probabilities of all populations belonging to *E.andevalensis*. K = 3, was calculated by cross-entropy scores.

Bayesian population assignment probabilities revealed a highly structured assemblage into three clusters in *E.andevalensis*. This number of clusters was the optimal aggrupation within the dataset following cross-entropy scores. The assemblage is consistent with geographical distribution of populations from São Domingos and Tinto1, which were the most homogeneous, and admixture with other clusters is nearly absent. These two populations corresponded with two of the clusters (green and red, respectively in Fig. 7). The third cluster (blue) was dominant in the population from Odiel, in which the other two clusters were also represented. Finally, all plants from Tinto2 population showed high levels of admixture mainly from red and blue clusters.

We also assessed population structure through pairwise F_ST_ comparisons. The results align with clustering analyses, providing additional insights. Notably, São Domingos stood out as a genetically distinct population (F_ST_ = 0.906; 0.8; 0.652, from Tinto1, Tinto2 and Odiel populations respectively). Spanish populations (Odiel, Tinto1, and Tinto2) showed a lower genetic differentiation, although F_ST_ values among populations were relatively high (Odiel-Tinto1, F_ST_ = 0.392; Tinto2-Tinto1: 0.466). Only the genetic differentiation between Odiel and Tinto2 was moderate low (Fst = 0.122).

The nucleotide diversity (π_i_) and heterozygosity (H_o_ and H_e_) in *E.andevalensis* exhibited significant variation among populations, with Odiel and Tinto2 ranking as the most diverse, in that order. Additionally, these two populations showed a lower F_IS_ index (Table 2). In contrast, São Domingos displayed an exceptionally low level of diversity, being more than eight times less nucleotide diverse than the Odiel population. Furthermore, São Domingos emerged as the population with a higher excess of homozygotes than expected (F_IS_ = 0.674).

**Table 2.** Summary diversity statistics of *E.andevalensis* populations calculated across sampling locations in which more than two samples were collected.

Population	N	H_o_	H_e_	F_IS_	π_i_
Odiel	11	0.155	0.289	0.464	0.219
São Domingos	16	0.028	0.087	0.674	0.025
Tinto 1	5	0.049	0.106	0.537	0.044
Tinto 2	4	0.148	0.247	0.401	0.163
**Total**	**36**	**0.083**	**0.169**	**0.508**	**0.10**

Nucleotide diversity (π_i_) and heterozygosity (H_o_ and H_e_) in *E.andevalensis* was in general similar to *E.mackayana* (Suppl. material 1: table S6). However, Odiel and Tinto2 populations were more diverse than those of *E.mackayana*, while Tinto1 and São Domingos are less. F_IS_ index was high in both species for the two species in all sampling areas, but higher in *E.andevalensis*.

## ﻿Discussion

This study provides evidence supporting the status of *Ericaandevalensis* as an accepted species, clearly distinct from its sister-group *E.mackayana*. Both morphological and genetic variability between species is much higher than among populations, and populations are highly structured in *E.andevalensis*, as previously found for *E.mackayana* (Fagúndez and Díaz-Tapia 2023). *Ericaandevalensis* and *E.mackayana* share a number of traits that are rare or unique in the northern heathers such as broad leaves, umbel-like terminal inflorescences and the presence of pluricellular glandular hairs in leaves and stems. Together with *E.tetralix* and *E.ciliaris*, they belong to a robust clade that exhibits further unique features such as pluricellular glandular indumentum or unrolled leaf margins, compared to other paleartic species (Bayer 1993; Nelson 2012; Mugrabi de Kuppler et al. 2015).

### ﻿Morphology and phenotypic analysis

The analysis of morphological traits provided reliable diagnostic characters for *E.mackayana* and *E.andevalensis* (Table 1, Figs 4, 5). These included the presence of some short hairs in the apex of the ovary near the insertion point of the style in *E.andevalensis*, which are absent in *E.mackayana*. This was previously stated by Bayer (1993), but not in the original description of *E.andevalensis*, in which it is described as having a glabrous ovary (Cabezudo and Rivera 1980). Variation in ovary hairiness is one of the most informative traits among species of the northern heathers, varying consistently between species even within closely related groups such as the *E.ciliaris-tetralix* clade (Nelson 2012). Hybridization among species of this group is recognized by the presence of hairs in the ovary, generally fewer and localized towards the apex as in E.×stuartii (*E.tetralix* × *E.mackayana*, Fagúndez 2006).

Other diagnostic characters related to leaf indumentum included the presence of short unicellular hairs in *E.andevalensis* which are nearly absent in *E.mackayana*, and long, pluricellular glandular trichomes in the two species but much longer in *E.mackayana*. Diagnostic differences in leaf hairiness are also reflected in the hairiness of other vegetative and non-vegetative organs, such as stems and flower pedicels (Cabezudo and Rivera 1980).

Leaf arrangement also contributed to species delimitation, as *E andevalensis* consistently shows 4-nate whorls of erect leaves. In *E.mackayana*, leaves are 4-6-nate and patent. Leaves of *E.andevalensis* are similar in size to those of *E.mackayana*, but narrower with more rolled-in margins. Remarkably, leaf length shows minimum contribution to PC1, but the highest weight in PC2, reflecting strong variation at the population level (Suppl. material 1: table S4). Leaf size is commonly described as a plant trait with high variation due to phenotypic plasticity (Stotz et al. 2022). This character may be informative to understand adaptation to stress factors or environmental constraints such as climate and soil condition in this group of heathers.

Other traits showed limited potential as diagnostic characters, particularly those related to reproductive biology. In *E.andevalensis*, flowers can be shorter and narrower, with a more pronounced style exertion. However, flower size and proportions can vary at different development stages. This variability is even more pronounced in ovary morphology and size (Suppl. material 1: table S3). *E.andevalensis* tends to have larger but flatter ovaries, but ovary size and shape depends on its development stage in transition towards fruit formation, which is difficult to assess. With regards to anther traits, *E.andevalensis* has darker and longer anthers, with a wider aperture, and a larger knob (a small protuberance in the anther) than *E.mackayana*, but these traits showed high overlapping values (Suppl. material 1: table S3).

### ﻿Genetic identity of *Ericaandevalensis*

Phylogenetic analyses show that both *Ericamackayana* and *E.andevalensis* are well supported monophyletic groups. Reciprocal monophyly is accepted as one of the most important lines of evidence on species delimitation (Moritz 1994; Mehta et al. 2019). Genetic distance has also been used extensively in species delimitation. We found that genetic distance was >4-fold higher between the species *E.andevalensis* and *E.mackayana* than among their populations. A much greater distance between species compared to populations supports their consideration as different species, in line with Birky et al. (2010) and Dellicour and Flot (2015). A combined framework for species delimitation, incorporating both phylogenetic tree-based approaches and genetic distance analysis, has been applied across various taxonomic groups (e. g. Bradley and Baker 2001; Meudt et al. 2009; Del Prado et al. 2011; Goicoechea et al. 2012).

The much greater branch lengths in *E.andevalensis* than in *E.mackayana* (Fig. 6), are consistent with results from phylogenetic analysis of plastid and nuclear ribosomal DNA sequences in Mugrabi de Kuppler et al. (2015) and could reflect a consistently faster rate of sequence divergence in *E.andevalensis*. Smaller population sizes resulting from edaphic specialization over time could contribute to this phenomenon. Small population size has been correlated with fast evolution empirically in many groups (Lanfear et al. 2014). There is potential for further exploration of the relation between genomic processes and enhanced ability of *E.andevalensis* to adapt to extreme environments. *Ericaandevalensis*, has been extensively studied for its metal-tolerant characteristics, thriving in heavily polluted soils with high concentrations of Cu, Ni, or F (e.g. Márquez et al. 2005; Rossini-Oliva et al. 2018). Its close relative, *E.mackayana*, can grow in a wider range of soils, even in serpentine soils with high levels of heavy metals, (e.g. A Capelada mountain range in NW Spain) and can inhabit areas with pH as low as 3 (Webb 1955; Fagúndez and Pontevedra-Pombal 2022), so may also exhibit pyritic-soil tolerance.

### ﻿Intraspecific variation in *Ericaandevalensis*

Two of the studied populations of *E.andevalensis* showed low support for internal nodes in the phylogeny, but the population structure analysis showed low levels of admixture, meaning that *E.andevalensis* populations are highly structured. The genetic differentiation among populations separated by less than 85 km is higher than in the entire distribution area analyzed for the related species *E.mackayana* (Fagúndez and Díaz-Tapia 2023). A high differentiation among populations is typically found in taxa with a highly fragmented distribution, as in soil endemic plants (Leimu and Mutikainen 2005; Nistelberger et al. 2015). The large genetic divergence between Tinto1 and Tinto2, separated by only 7.5 km and initially considered as a single population, was a surprising finding. We found a lower proximity due to river section (medium *vs* low river course) than that of river identity (Odiel *vs* Tinto).

Soil endemic plants usually have low genetic diversity, but their F_IS_ index clearly diverge between different plants groups (e. g. Barbará et al. 2007; Wang et al. 2017; Nagasawa et al. 2019). It has been suggested that species traits such as self-compatibility or clonality might impact the F_IS_ index (Lavor et al. 2014), thus comparing soil endemic species F_IS_ values with related taxa with broader ranges can be interesting. Compared to its close relative *E.mackayana*, *E.andevalensis* had higher F_IS_ index values, and genetic diversity was highly variable among *E.andevalensis* populations. São Domingos population results show the highest F_IS_ value and lowest genetic diversity of all populations of both species, in line with results from the *E.andevalensis* microsatellites’ analyses (Bandeira de Albuquerque et al. 2008), and probably reflecting a founder effect. This population is at the edge of the species distribution range, entirely located in an abandoned mining area (Figs 1C, 2).

The origin and migration history of different *Erica* species are a subject of debate, especially when there is a potential transportation linked to human activities (Fagúndez and Díaz-Tapia 2023; Skeffington and Scott 2023). Nelson et al. (1985) suggested that mining populations of *E.andevalensis* might not be native, originating with the commencement of mining activity in the area, estimated by Tornos et al. (2000) to be more than 5000 years ago. This hypothesis aligns with the low genetic diversity of São Domingos, but Bandeira de Albuquerque et al. (2008) found high genetic diversity in other populations in Huelva linked to mining activities. Nevertheless, the creation of new niches by humans (highly toxic damp mines), coupled with an increased concentration of heavy metals in rivers, could have facilitated the establishment of larger populations beyond their natural limits and possibly a recent migration between previously unconnected populations. This is consistent with the moderate connection between populations in the lower Odiel and Tinto basins and could explain higher genetic diversity in these populations and slightly lower F_IS_ index. The small size of the seeds which are produced in large numbers makes them easily dispersed by animals, wind and other vectors including humans (Aparicio and García-Martín 1996; Fagúndez and Izco 2004; Fagúndez and Izco 2010).

### ﻿Implications for conservation

Clarifying cryptic or poorly understood taxa and species delimitation is needed for the design of conservation policies and actions, especially in large genera such as *Erica* (Pirie et al. 2022). Verification of specific status should give stronger support to the natural value of the Iberian Pyrite Belt, which is partly a protected area in Spain (BOJA 2005). *Ericaandevalensis* is legally considered an endangered species, classified as vulnerable (VU), but only in Spain. The low genetic diversity and singularity of the Portuguese population, both phenotypical and genotypical, coupled with its presence in a very restricted area, should justify the consideration of *E.andevalensis* as an endangered species in Portugal as well, as proposed in Carapeto et al. (2020).

Conservation strategies for *E.andevalensis* should protect a variety of populations in different river basins and sections, and research evaluating its use for restoration should consider infraspecific variability and special issues of working with metallophyte species. For instance, habitat decontamination of heavy metal polluted river shores needs to be carefully considered, as it may have a negative effect in local populations of *E.andevalensis* (Márquez et al. 2005). Recommended conservation actions of metallophytes endemic species usually include prohibiting new mining activities in areas (Whiting et al. 2004). This recommendation was also made to conserve *Ericaandevalensis* populations (Márquez et al. 2005). However, certain metallophytes may benefit from human perturbations in the long-term, potentially leading to an increase in their populations (Faucon et al. 2011), and this may be the case for *E.andevalensis*. Many conservation studies on metallophytes focus on areas where mining has been intensive but only during the last century. Our study, by contrast, shows the genetic and dispersal effects of long-term mining (>5000 years BP) in an endemic metallophyte species.

There is potential for use of *E.andevalensis* in bioremediation of polluted soils (Abreu et al. 2008; Monaci et al. 2010; Pérez-López et al. 2014). Differences with regards to soil preference between Spanish and Portuguese populations (Pérez-López et al. 2014), suggest a potential variability in bioremediation performance at the population level. These ecological differences, also reflected in genetics, should be considered in planning habitat restoration.

## ﻿Additional information

### Author ORCIDs

Iván Rodríguez-Buján https://orcid.org/0000-0002-9869-4033

Pilar Díaz-Tapia https://orcid.org/0000-0003-4680-4867

Jaime Fagúndez https://orcid.org/0000-0001-6605-7278

### Data availability

All of the data that support the findings of this study are available in the main text or Supplementary Information.

## Supplementary materials

Supplementary material 1 Supplementary tables Data type docx Explanation note **table S1**: Information on the populations of the studied species *Ericamackayana* and *E.andevalensis*, and one population of *E.tetralix* used as the outgroup. N_1_: number of individuals used in the genetic analyses, based on having <50% of missing data in population structure analysis. N_2_: number of individuals used in the morphological analyses. **table S2**: Morphological traits measured in plants of *Ericamackayana* and *E.andevalensis*, acronym names and methodology. QN = Quantitative trait, O = Ordinal trait, B = Binary trait. N = Number of measures per specimen. **table S3**: Results of univariant tests between *Ericamackayana* and *E.andevalensis*, including the arithmetic mean and range for each of the morphological traits named as in Fig. 1 and Suppl. material 1: table S2. *E.mack* = *E.mackayana*. *E.andev*. = *E.andevalensis*. * = p-value < 0.05, **= p-value < 0.01, *** = p-value < 0.001. **table S4**: Percentage of contribution of each morphological trait included in the PCA to the first two components (PC1 and PC2), named as in Fig. 1 and Suppl. material 1: table S2, ordered by their contribution to PC1. **table S5**: Distance matrix for the studied populations of *Ericamackayana* and *E.andevalensis*. Pairwise Tamura and Nei distance (1993) calculated upon MEGA 11. **table S6**: Summary of the genetic diversity statistics of *E.andevalensis* and *E.mackayana* populations. Statistics for populations with less than three individuals were not included. https://binary.pensoft.net/file/1085798This dataset is made available under the Open Database License (http://opendatacommons.org/licenses/odbl/1.0/). The Open Database License (ODbL) is a license agreement intended to allow users to freely share, modify, and use this Dataset while maintaining this same freedom for others, provided that the original source and author(s) are credited.Iván Rodríguez-Buján, Pilar Díaz-Tapia, Jaime Fagúndez
