# Supplementary material for: ﻿Genetic and morphological evidence support the specific status of the endemic Ericaandevalensis (Ericales, Ericaceae)
Source: PhytoKeys. 2024 Jul 5;244:57–76. doi: 10.3897/phytokeys.244.120914 (PMC11245639; doi:10.3897/phytokeys.244.120914)
Supplement: Supplementary material 1 — Supplementary tables [file phytokeys-244-057_article-120914__-s001.docx]

*Supplementary materials of:* **Genetic and morphological evidence support the specific status of the endemic *Erica andevalensis*** *by Rodríguez-Buján et al.*

**Table S1:** Information on the populations of the studied species *Erica mackayana* and *E. andevalensis*, and one population of *E. tetralix* used as the outgroup. N_1_: number of individuals used in the genetic analyses, based on having <50% of missing data in population structure analysis. N_2_: number of individuals used in the morphological analyses.

| Species | Population | Country | Collector | Year | Latitude (⁰N) | Longitude (⁰W) | Elevation (m) | N_1_ | N_2_ |
| --- | --- | --- | --- | --- | --- | --- | --- | --- | --- |
| *E. andevalensis* | Tinto1 | Spain | J. Fagúndez | 2021 | 37.42 | 6.61 | 33 | 5 | 5 |
| *E. andevalensis* | Tinto2 | Spain | J. Fagúndez | 2021 | 37.37 | 6.67 | 13 | 4 | 5 |
| *E. andevalensis* | Odiel | Spain | J. Fagúndez | 2021 | 37.60 | 6.84 | 56 | 11 | 12 |
| *E. andevalensis* | São Domingos | Portugal | J. Fagúndez | 2021 | 37.66 | 7.50 | 124 | 16 | 16 |
| *E. mackayana* | Pimiango | Spain | J. Fagúndez | 2016 | 43.39 | 4.54 | 122 | 2 | 3 |
| *E. mackayana* | Peñas | Spain | J. Fagúndez | 2016 | 43.65 | 5.85 | 98 | 2 | 3 |
| *E. mackayana* | Espina | Spain | J. Fagúndez | 2016 | 43.39 | 6.31 | 688 | 13 | 15 |
| *E. mackayana* | Bustantigo | Spain | J Fagúndez, A Montañés | 2016 | 43.33 | 6.71 | 1011 | 7 | 8 |
| *E. mackayana* | Xistral | Spain | J. Fagúndez | 2016 | 43.43 | 7.48 | 753 | 8 | 8 |
| *E. mackayana* | Loba | Spain | J. Fagúndez | 2015 | 43.28 | 7.94 | 632 | 10 | 12 |
| *E. mackayana* | Donegal | Ireland | M. Sheehy Skeffington & R. Shepard | 2013-2015 | 55.03 | 8.16 | 57 | 3 | 4 |
| *E. mackayana* | Mayo | Ireland | M. Sheehy Skeffington | 2013-2014 | 54.11 | 9.52 | 85 | 3 | 7 |
| *E. mackayana* | Galway | Ireland | M. Sheehy Skeffington | 2013-2014-2015 | 53.43 | 9.89 | 22 | 9 | 13 |
| *E. mackayana* | Carna | Ireland | M. Sheehy Skeffington | 2015 | 53.32 | 9.81 | 20 | 1 | 2 |
| *E. mackayana* | Kerry | Ireland | M. Sheehy Skeffington | 2013-2014 | 51.93 | 10.07 | 120 | 3 | 4 |
| *E. tetralix* | Mées | France | J. Fagúndez | 2022 | 43.70 | 1.13 | 30 | 1 | 0 |

**Table S2:** Morphological traits measured in plants of *Erica mackayana* and *E. andevalensis*, acronym names and methodology. QN= Quantitative trait, O = Ordinal trait, B = Binary trait. N = Number of measures per specimen.

| Number | Character | Acronym | Methods | Type | N | Units / Coding | Accuracy | Additional explanation |
| --- | --- | --- | --- | --- | --- | --- | --- | --- |
|  | **Leaf** |  |  |  |  |  |  |  |
| 1 | Leaf length | LF | Digital image sheet | QN | >5 | mm | 40 µm |  |
| 2 | Leaf width | LW | Digital image sheet | QN | >5 | mm | 40 µm |  |
| 3 | Leaf width to length ratio (2/1) | LWLF | Calculation | QN | >5 | - |  |  |
| 4 | Petiole length | LP | Digital image sheet | QN | >5 | mm | 40 µm |  |
| 5 | Length petiole to length ratio (4/1) | LPLF | Calculation | QN | >5 | - |  |  |
| 6 | Leaf rolliness | LR | Digital image sheet | O | 1 | 1-5 | 40 µm | 1 = Leaf margin rolled almost completely. Only a narrow line of the abaxial surface is exposed.  2 = The abaxial face is visible around 25% compared to the adaxial face.  3 = The abaxial face is visible around 50% compared to the adaxial face.  4 = The abaxial face is visible around 75% compared to the adaxial face.  5 = Absence of rolling |
| 7 | Leaf insertion angle | LIA | Digital image sheet | O | 1 | 1-5 | - | 1 = Erect  2 = Partly erect  3 = Intermediate  4 = Partly patent  5 = Patent |
| 8 | Density of leaf whorls in a flowering branch | LVD | Digital image sheet | QN | 1 | Whorls/  branch length | 40 µm | Number of leaf whorls in a flowering branch up to the first branch ramification / Length of the same branch |
| 9 | Length of glandular hair | LSG | Optical microscope | QN | 1 | µm | 10 µm |  |
| 10 | Presence of non-glandular hairs | LNR | Stereo microscope | B | 1 | 0-1 | - |  |
| 11 | Presence of more than 2 rows of glandular hair | LS2G | Stereo microscope | B | 1 | 0-1 | - |  |
| 12 | Presence of at least one leaf whorl with 5 leaves | L5 | Digital image sheet | B | 1 | 0-1 | - | 0 = Absence  1 = Presence |
|  | **Anthers** |  |  |  |  |  |  |  |
| 13 | Anther length | AL | Stereo microscope | QN | 1-4 | µm | 5 µm |  |
| 14 | Anther pore length | AO | Stereo microscope | QN | 1-4 | µm | 5 µm |  |
| 15 | Anther appendix length | AAL | Stereo microscope | QN | 1-4 | µm | 5 µm |  |
| 16 | Anther appendix curvature | AAC | Stereo microscope | QN | 1-4 | 1-5 | 5 µm | 1 = Straight  2 = Slightly curved  3 = Forming a C-shaped curve  4 = Forming a curve close to closing itself  5= Forming a curve that closes itself |
| 17 | Anther pore length to anther length ratio (14/13) | AOAL | Stereo microscope | QN | 1-4 | - | 5 µm |  |
| 18 | Anther colour | AC | Stereo microscope | O | 1 | 1-5 | - | 1 = Black  2 = Almost black  3 = Dark Brown  4 = Intermediate  5 = Light brown |
| 19 | Size of the knob of the anther | AK | Stereo microscope | O | 1 | 1-5 | - | 1 = Absence  3 = Presence of a small knob  5 = Very prominent |
|  | **Ovary** |  |  |  |  |  |  |  |
| 20 | Ovary length | OL | Stereo microscope | QN | 1 | µm | 5 µm |  |
| 21 | Ovary width | OW | Stereo microscope | QN | 1 | µm | 5 µm |  |
| 22 | Nectary size | ON | Stereo microscope | QN | 1 | µm | 5 µm |  |
| 23 | Ovary hairiness | OH | Stereo microscope | O | 1 | 0-5 | - | 0 = Absence  1 = No more than 2 or 3 hairs in the apex extreme  2 = A few hairs extending slightly beyond the apex of the ovary and more numerous. The ovary continues to seem glabrescent.  3= Presence of sparse hairs throughout ovarium  4=Ovary hairy, but with gaps  5=Ovary fully hairy |
| 24 | Ovary width to length ratio (21/20) | OWOL | Stereo microscope | QN | 1 | - | - |  |
| 25 | Ovary nectary size to length ratio (22/20) | ONOL | Stereo microscope | QN | 1 | - | - |  |
|  | **Flowers** |  |  |  |  |  |  |  |
| 26 | Corolla length | FL | Digital image sheet | QN | >5 | mm | 40 µm |  |
| 27 | Corolla width | FW | Digital image sheet | QN | >5 | mm | 40 µm |  |
| 28 | Width of the corolla opening | FWC | Digital image sheet | QN | >5 | mm | 40 µm |  |
| 29 | Style flower exertion | FS | Digital image sheet | QN | >5 | mm | 40 µm |  |
| 30 | Corolla width to length ratio (27/26) | FWFL | Calculation | QN | >5 | - | - |  |
| 31 | Width of the corolla opening to flower length ratio (28/26) | FWCFL | Calculation | QN | >5 | - | - |  |
| 32 | Flowers per inflorescence | FI | Digital image sheet | QN | 1 | count | - |  |
| 33 | Pedicel hairiness | FHP | Digital image sheet | O | 1 | 1-5 | 40 µm | 1 = Hairs are so small and/or scarce that pedicel it’s difficult to individualize it  2 = Some hairs are clearly individualized, but they are very scarce  3 =Intermediate  4 = Hairs cover most part pedicel but not totally  5 = Full of wooly long hairs |
| 34 | Pedicel length | FLP | Digital image sheet | O | 1 | 1-7 | 40 µm | 1= < 2 mm  2 = 2-2.5 mm  3 = 2.5-3 mm  4 = 3-3.5 mm  5 = 3.5-4 mm  6 = 4-4.5 mm  7 = > 4.5 mm |
|  | **Stem** |  |  |  |  |  |  |  |
| 35 | Longest hair on a branch | BLH | Digital image sheet | QN | 1 | µm | 40 µm |  |

**Table S3:** Results of univariant tests between *Erica mackayana* and *E. andevalensis*, including the arithmetic mean and range for each of the morphological traits named as in Figure 1 and Table S2. *E. mack* = *E. mackayana*. *E. andev. = E. andevalensis*. * = p-value < 0.05, **= p-value < 0.01, *** = p-value < 0.001.

| Trait | Units | Type of test | *E. mack* mean | *E. mack*  range | *E. andev.*  mean | *E. andev.* Range | p-value |
| --- | --- | --- | --- | --- | --- | --- | --- |
| LF | mm | t-Student | 2.944 | 1.749 - 5.371 | 2.929 | 1.662 - 3.650 | 0.903 |
| LW | mm | t-Student | 0.931 | 0.538 - 1.440 | 1.179 | 0.780 - 1.715 | *** |
| LWLF | - | t-Student | 0.344 | 0.213 - 0.807 | 0.405 | 0.298 - 0.494 | *** |
| LP | mm | Wilcoxon | 0.651 | 0.369 - 1.023 | 0.721 | 0.535 - 0.868 | *** |
| LPLF | - | t-Student | 0.234 | 0.137 - 0.793 | 0.251 | 0.202 - 0.324 | 0.180 |
| LR | 1-5 | Chi-squared | 2.937 | 2 - 5 | 1 | - | *** |
| LIA | 1-5 | Chi-squared | 3.696 | 2 - 5 | 2.395 | 1 - 4 | *** |
| LVD | Whorls/  branch length | t-Student | 0.535 | 0.191 - 1.016 | 0.686 | 0.353 - 1.122 | *** |
| LSG | µm | Wilcoxon | 562.025 | 425 - 775 | 136.448 | 50 - 280 | *** |
| LNR | 0-1 | Fisher | 0 | - | 0.839 | 0 - 1 | *** |
| LS2G | 0-1 | Fisher | 0 | - | 0.773 | 0 - 1 | *** |
| L5 | 0-1 | Fisher | 0.958 | 0 - 1 | 0 | - | *** |
| AL | µm | Wilcoxon | 0.814 | 0.648 - 1.047 | 0.925 | 0.794 - 1.017 | *** |
| AO | µm | Wilcoxon | 0.448 | 0.279 - 0.585 | 0.527 | 0.406 - 0.607 | *** |
| AAL | µm | Wilcoxon | 0.770 | 0.429 | 0.571 | 0.430 - 0.687 | *** |
| AAC | 1-5 | Chi-squared | 2.918 | 1 - 5 | 2.5 | 1 - 4 | * |
| AOAL | - | Wilcoxon | 0.548 | 0.422 - 0.676 | 0.570 | 0.504 - 0.630 | * |
| AC | 1-5 | Chi-squared | 2.041 | 1 - 5 | 1.447 | 1 - 3 | ** |
| AK | 1-5 | Chi-squared | 3.080 | 1 - 5 | 4.789 | 3 - 5 | *** |
| OL | µm | Wilcoxon | 1.069 | 0.774 - 1.647 | 1.190 | 0.843 - 1.601 | * |
| OW | µm | Wilcoxon | 1.015 | 0.663 - 1.763 | 1.369 | 0.798 - 2.077 | *** |
| ON | µm | Wilcoxon | 0.205 | 0.110 - 0.418 | 0.209 | 0.137 - 0.314 | 0.751 |
| OH | 0-5 | Chi-squared | 0 | - | 1.083 | 1 - 2 | *** |
| OWOL | - | Wilcoxon | 1.103 | 0.633 - 1.763 | 0.879 | 0.717 - 1.182 | *** |
| ONOL | - | Wilcoxon | 0.205 | 0.110 - 0.418 | 0.181 | 0.117 - 0.263 | 0.144 |
| FL | mm | Wilcoxon | 5.175 | 4.064 - 6.728 | 4.576 | 3.941 - 5.272 | *** |
| FW | mm | Wilcoxon | 2.973 | 2.345 - 3.781 | 2.838 | 2.589 - 3.187 | * |
| FWC | mm | Wilcoxon | 1.556 | 1.042 - 2.047 | 1.508 | 1.042 - 2.047 | 0.197 |
| FS | mm | Wilcoxon | 0.587 | 0 - 1.114 | 0.967 | 0.581 - 1.373 | *** |
| FWFL | - | Wilcoxon | 0.578 | 0.442 - 0.692 | 0.623 | 0.547 - 0.692 | *** |
| FWCFL | - | Wilcoxon | 0.527 | 0.376 - 0.691 | 0.532 | 0.437 - 0.638 | 0.618 |
| FI | count | Wilcoxon | 10.937 | 4 - 21 | 11.842 | 7 - 16 | 0.119 |
| FHP | 1-5 | Chi-squared | 2.500 | 1 - 3 | 1.105 | 1 - 2 | *** |
| FLP | 1-7 | Chi-squared | 5.404 | 3 - 7 | 4.639 | 2 - 7 | * |
| BLH | µm | Wilcoxon | 0.986 | 0.674 - 1.450 | 0.333 | 0.120 – 0.572 | *** |

**Table S4:** Percentage of contribution of each morphological trait included in the PCA to the first two components (PC1 and PC2), named as in Figure 1 and Table S2, ordered by their contribution to PC1.

| **Trait** | **Contribution to PC1** | **Contribution to PC2** | **Contribution to PC1+PC2** |
| --- | --- | --- | --- |
| OH | 8.584 | 0.000 | 0.921 |
| LSG | 8.173 | 0.103 | 0.881 |
| L5 | 7.890 | 0.011 | 0.847 |
| LS2G | 7.452 | 0.021 | 0.800 |
| LNR | 7.412 | 0.187 | 0.803 |
| BLH | 7.369 | 0.246 | 0.801 |
| LR | 6.506 | 0.125 | 0.703 |
| FHP | 5.703 | 0.607 | 0.637 |
| FS | 4.451 | 0.330 | 0.491 |
| AL | 3.797 | 2.922 | 0.530 |
| LIA | 3.493 | 0.335 | 0.389 |
| AO | 3.355 | 3.242 | 0.496 |
| FL | 2.982 | 4.679 | 0.516 |
| OW | 2.955 | 4.760 | 0.517 |
| LW | 2.938 | 2.668 | 0.427 |
| AK | 2.379 | 0.190 | 0.263 |
| OWOL | 2.193 | 2.942 | 0.359 |
| FWFL | 1.888 | 0.517 | 0.224 |
| LVD | 1.670 | 5.561 | 0.413 |
| LWLF | 1.307 | 2.192 | 0.232 |
| AAL | 1.148 | 0.376 | 0.139 |
| AC | 1.017 | 5.415 | 0.336 |
| FLP | 0.971 | 9.191 | 0.490 |
| OL | 0.768 | 1.552 | 0.148 |
| LP | 0.742 | 8.742 | 0.447 |
| AAC | 0.723 | 0.918 | 0.116 |
| FW | 0.638 | 9.984 | 0.488 |
| LPLF | 0.516 | 0.129 | 0.014 |
| AOAL | 0.488 | 1.250 | 0.105 |
| ONOL | 0.371 | 1.945 | 0.121 |
| FWC | 0.328 | 6.416 | 0.305 |
| FI | 0.212 | 4.172 | 0.198 |
| LF | 0.006 | 10.707 | 0.450 |
| FWCFL | 0.004 | 0.085 | 0.004 |
| ON | 0.003 | 7.476 | 0.314 |

**Table S5:** Distance matrix for the studied populations of *Erica mackayana* and *E. andevalensis*. Pairwise Tamura & Nei distance (1993) calculated upon MEGA 11.

| Species |  | *Erica andevalensis* | | | | *Erica mackayana* | | | | | | | |
| --- | --- | --- | --- | --- | --- | --- | --- | --- | --- | --- | --- | --- | --- |
|  | Populations | Tinto1 | Tinto2 | Odiel | São Domingos | Espina | Bustantigo | Xistral | Loba | Donegal | Mayo | Galway | Kerry |
| *Erica andevalensis* | Tinto1 |  | 0.137 | 0.214 | 0.417 | 1.351 | 1.375 | 1.136 | 1.018 | 1.112 | 1.300 | 1.156 | 0.945 |
|  | Tinto2 |  |  | 0.144 | 0.346 | 1.232 | 1.277 | 1.136 | 1.004 | 1.085 | 1.211 | 1.189 | 0.957 |
|  | Odiel |  |  |  | 0.351 | 1.299 | 1.302 | 1.117 | 1.045 | 1.036 | 1.108 | 1.102 | 1.019 |
|  | São Domingos |  |  |  |  | 1.223 | 1.216 | 1.152 | 1.050 | 1.149 | 1.206 | 1.119 | 1.038 |
| *Erica mackayana* | Espina |  |  |  |  |  | 0.224 | 0.265 | 0.246 | 0.304 | 0.237 | 0.352 | 0.290 |
|  | Bustantigo |  |  |  |  |  |  | 0.256 | 0.246 | 0.299 | 0.252 | 0.352 | 0.285 |
|  | Xistral |  |  |  |  |  |  |  | 0.237 | 0.273 | 0.286 | 0.326 | 0.270 |
|  | Loba |  |  |  |  |  |  |  |  | 0.265 | 0.291 | 0.328 | 0.260 |
|  | Donegal |  |  |  |  |  |  |  |  |  | 0.331 | 0.312 | 0.264 |
|  | Mayo |  |  |  |  |  |  |  |  |  |  | 0.409 | 0.312 |
|  | Galway |  |  |  |  |  |  |  |  |  |  |  | 0.359 |
|  | Kerry |  |  |  |  |  |  |  |  |  |  |  |  |

**Table S6:** Summary of the genetic diversity statistics of *E. andevalensis* and *E. mackayana* populations. Statistics for populations with less than three individuals were not included.

|  | Population | N | H_o_ | H_e_ | F_IS_ | π_i_ |
| --- | --- | --- | --- | --- | --- | --- |
| *Erica mackayana* | Bustantigo | 7 | 0.108 | 0.181 | 0.403 | 0.142 |
|  | Carna | 1 | - | - | - | - |
|  | Donegal | 3 | 0.058 | 0.083 | 0.303 | 0.055 |
|  | Espina | 13 | 0.065 | 0.083 | 0.225 | 0.142 |
|  | Galway | 9 | 0.042 | 0.061 | 0.308 | 0.087 |
|  | Kerry | 3 | 0.106 | 0.176 | 0.400 | 0.071 |
|  | Loba | 10 | 0.072 | 0.086 | 0.168 | 0.147 |
|  | Mayo | 4 | 0.121 | 0.141 | 0.146 | 0.071 |
|  | Peñas | 2 | - | - | - | - |
|  | Pimiango | 2 | - | - | - | - |
|  | Xistral | 8 | 0.108 | 0.181 | 0.403 | 0.147 |
|  | **Total** | **62** | **0.094** | **0.149** | **0.355** | **0.121** |
| *Erica andevalensis* | Odiel | 11 | 0.155 | 0.289 | 0.464 | 0.219 |
|  | São Domingos | 16 | 0.028 | 0.087 | 0.674 | 0.025 |
|  | Tinto 1 | 5 | 0.049 | 0.106 | 0.537 | 0.044 |
|  | Tinto 2 | 4 | 0.148 | 0.247 | 0.401 | 0.163 |
|  | **Total** | **36** | **0.083** | **0.169** | **0.508** | **0.10** |
